# A genomic island present along the bacterial chromosome of the *Parachlamydiaceae *UWE25, an obligate amoebal endosymbiont, encodes a potentially functional F-like conjugative DNA transfer system

**DOI:** 10.1186/1471-2180-4-48

**Published:** 2004-12-22

**Authors:** Gilbert Greub, François Collyn, Lionel Guy, Claude-Alain Roten

**Affiliations:** 1 Center for Research on Intracellular Bacteria, Institute of Microbiology, Faculty of Biology and Medicine, University of Lausanne, Lausanne, Switzerland; 2 E0364 Inserm, Etude des Interactions Cellulaires et Moléculaires des Bactéries Pathogènes avec l'Hôte, Institut de Biologie de Lille & Faculté de Médecine Henri Warembourg, Université de Lille II, Lille, France; 3 Department of Fundamental Microbiology, Faculty of Biology and Medicine, University of Lausanne, Lausanne, Switzerland

## Abstract

**Background:**

The genome of *Protochlamydia amoebophila *UWE25, a *Parachlamydia*-related endosymbiont of free-living amoebae, was recently published, providing the opportunity to search for genomic islands (GIs).

**Results:**

On the residual cumulative G+C content curve, a G+C-rich 19-kb region was observed. This sequence is part of a 100-kb chromosome region, containing 100 highly co-oriented ORFs, flanked by two 17-bp direct repeats. Two identical *gly-tRNA *genes in tandem are present at the proximal end of this genetic element. Several mobility genes encoding transposases and bacteriophage-related proteins are located within this chromosome region. Thus, this region largely fulfills the criteria of GIs. The G+C content analysis shows that several modules compose this GI. Surprisingly, one of them encodes all genes essential for F-like conjugative DNA transfer (*traF*, *traG*, *traH*, *traN*, *traU*, *traW*, and *trbC*), involved in sex pilus retraction and mating pair stabilization, strongly suggesting that, similarly to the other F-like operons, the parachlamydial *tra *unit is devoted to DNA transfer. A close relatedness of this *tra *unit to F-like *tra *operons involved in conjugative transfer is confirmed by phylogenetic analyses performed on concatenated genes and gene order conservation. These analyses and that of *gly-tRNA *distribution in 140 GIs suggest a proteobacterial origin of the parachlamydial *tra *unit.

**Conclusions:**

A GI of the UWE25 chromosome encodes a potentially functional F-like DNA conjugative system. This is the first hint of a putative conjugative system in chlamydiae. Conjugation most probably occurs within free-living amoebae, that may contain hundreds of *Parachlamydia *bacteria tightly packed in vacuoles. Such a conjugative system might be involved in DNA transfer between internalized bacteria. Since this system is absent from the sequenced genomes of *Chlamydiaceae*, we hypothesize that it was acquired after the divergence between *Parachlamydiaceae *and *Chlamydiaceae*, when the *Parachlamydia*-related symbiont was an intracellular bacteria. It suggests that this heterologous DNA was acquired from a phylogenetically-distant bacteria sharing an amoebal vacuole. Since *Parachlamydiaceae *are emerging agents of pneumonia, this GI might be involved in pathogenicity. In future, conjugative systems might be developed as genetic tools for *Chlamydiales*.

## Background

First described in 1997, *Parachlamydia acanthamoebae *is an obligate intracellular bacterium naturally infecting free-living amoebae [1,2]. It was isolated from *Acanthamoeba *spp. recovered from the nasal mucosa of healthy volunteers [1]. Later, additional strains of *Parachlamydiaceae *have been found within about 5% of *Acanthamoeba *spp. and once within *Hartmanella vermiformis *[2,3]. The 16S rRNA sequences of these *Parachlamydiaceae *are about 14% different from those of both genera *Chlamydophila *and *Chlamydia *[2,3]. Since the 16S rRNA sequence difference between *Chlamydophila *sp. and *Chlamydia *sp. is 6% only, it clearly appears that the speciation between the two latter occurred after the divergence between *Parachlamydiaceae *and *Chlamydiaceae*.

Like other *Chlamydiales*, *Parachlamydiaceae *can present two developmental stages: the reticulate body, a metabolically active dividing form, and the elementary body, an infective stage; the crescent body is another infective form, not observed in *Chlamydiaceae *[4]. Differentiation of the infective stages in reticulate bodies and multiplication of the latter were recently shown to occur within amoebal vacuoles, that may contain hundreds of bacteria [4]. Depending on the symbiotic/pathogenic relationships prevailing between both organisms, the escape of the bacteria from the amoeba may occur either by the release of secreted vesicles or by the lysis of the host [4].

There is a growing evidence of the human pathogenicity of *Parachlamydiaceae *[2]. For instance, positive *Parachlamydia *serologies were shown to be associated with a febrile epidemic [5], community-acquired pneumonia [6], and inhalation pneumonia [7]. The role of *Parachlamydia*-related bacteria as agents of inhalation pneumonia is further suggested by the temperature-dependent release of the bacteria from their amoebal reservoir [8]. PCR amplification of parachlamydial DNA from monocytes, sputa and bronchoalveolar lavages collected from patients suffering of bronchitis or pneumonia also supports the pathogenic potential of *Parachlamydia *[9-12]. The survival of these *Chlamydia*-like organisms within human macrophages [13] is an additional hint of parachlamydial pathogenicity.

Horn *et al*. [14], by sequencing and annotating the whole genome of the *Parachlamydia*-related UWE25 contributed much to the understanding of the evolution of chlamydiae. Indeed, they demonstrated that major virulence mechanisms of *Chlamydiaceae *such as the Type Three Secretion System (TTSS) and the Chlamydial Protease-like Activity Factor (CPAF) are also encoded by the chromosome of the evolutionary early-branching *Parachlamydiaceae *UWE25. Genome analysis of the parachlamydial endosymbiont also identified Open Reading Frames (ORFs) homologous to Type Four Secretion Systems (TFSS) and characterized by a high G+C content, suggesting that they result from an horizontal transfer. Based on their annotation revealing the apparent absence of genes necessary for DNA transfer, Horn *et al*. [14] proposed that this TFSS was involved in protein export but not in DNA transfer.

To date, numerous genomic islands (GIs) were already identified along whole chromosomal sequences of various bacterial species. For instance, 140 GIs are described in the Islander database, including GIs of proteobacteria, firmicutes, actinobacteria and cyanobacteria [15]. Thus, we wondered whether any GIs were located along the bacterial chromosome of the amoebal endosymbiont UWE25.

GIs are genetic elements which length vary from 10 to 200 kb and are inserted in a chromosome after a lateral transfer occurring, in some instances, between phylogenetically-distant microorganisms. Their heterologous origins are generally evidenced by a G+C content different from that of the remaining bacterial chromosome and by the presence of various mobility genes (i.e. involved in transposition, transduction or conjugative transfer), that are occasionally source of GI instability [16,17]. They are often flanked by particular DNA sequences, such as direct repeats or insertion sequences. Moreover, tRNA *loci *are generally used as insertion sites by GIs for their chromosomal integration [16-18]. Since no genetic tools are available for the study of this obligate intracellular bacteria, a bioinformatic approach was chosen to locate putative GIs.

## Results

### A genomic island is present in the genome of UWE25

**Figure 1:**
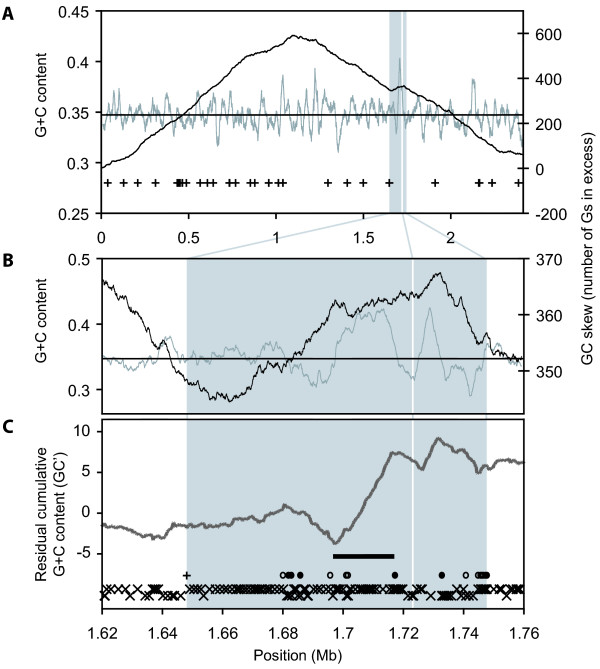
The genomic island (GI) present in the chromosome of the endosymbiont UWE25. **(A) **Position of the GI on the UWE25 genome, a 100-kb region (grey area) delimited by two direct repeats (Table 1) at both ends and by two *gly-tRNA*s genes in tandem (all tRNAs genes are represented by '+') at its proximal end. A third copy of the direct repeat (Table 1) is indicated by a white line disrupting the grey area. The region is characterized by a different slope in the cumulative GC skew analysis (black curve) and by a higher G+C content (grey curve, windows of 20 kb, 0.1-kb step). The horizontal line indicates the genomic G+C content average. **(B) **Closer view of the 100-kb region (black curve, cumulative GC skew; grey curve, G+C content windows of 5 kb, 0.1-kb step; horizontal line, average genomic G+C content). **(C) **Residual cumulative G+C content (GC') and genomic features of the 100-kb GI. This region encompasses the region with the highest G+C content in the 20-kb windows analysis of the UWE25 genome. The position of genes is represented by an 'X' on the upper line if encoded on the positive strand, otherwise by an 'X' on the bottom line (For details, see Table in Supplementary Material 1). A large majority of genes are co-oriented in the genome region flanked by the direct repeats. The *tra *operon (thick line), present on this GI, exhibits a G+C content (40.0%) clearly higher than that of the whole genome (34.7%). The positions of transposases (open circles) and of phage-related genes (full circles) are indicated.

Using standard G+C content analyses of *Parachlamydia*-related UWE25 chromosome, we observed a G+C-rich region (Figure 1A and 1B), similar to that shown by Horn *et al*. [14]. Using the residual cumulative G+C content analysis adapted from the GC profile of Zhang and Zhang [19], we were able to precisely define a 19-kb region (Figure 1C). The presence of 17-bp direct repeats flanking a 100-kb chromosome region (1648 to 1748 kb, Table 1) that encompasses the 19-kb DNA sequence enabled us to define a new region composed of 100 ORFs (See additional data file 1 for the description of these genes and their location on the chromosome of UWE25). Interestingly, this 100-kb region is characterized by a higher level of local gene coorientation (75/100) than that characterizing the remaining of the genome (1015/1931, 52.6%, p < 0.001) and by a particular signature in the cumulative GC skew analysis. Two identical *gly-tRNA *genes in tandem are located at the proximal end of this 100-kb genetic element (Figure 1A,1C and Table 1). Several mobility genes (eight putative transposases, one recombinase and seven bacteriophage related-proteins) are encoded within the 100-kb region (Figure 1C, Table 1). Thus, this region largely fulfills the accepted criteria of GIs [16-18]. We termed this newly described GI "Pam100G" (*Protochlamydia amoebophila*, 100-kb, Gly-tRNA) according to the nomenclature used in the Islander database [15].

### Mosaicism of the 100-kb genomic island

 Interestingly, this GI can be divided into clearly distinct regions, according to their G+C content (Figure 1B, Table 2). The residual cumulative G+C content analysis highlights a modular structure with different slopes, each   linear segment indicates that genes of this unit present a rather constant local G+C content (Figure 1C). A positive or a negative slope would indicate that   each block of genes presents a G+C content higher or lower that of the UWE25 chromosome, respectively. 

The first module begins with a direct repeat and two identical *gly-tRNAs *in tandem. Composed of 28 ORFs, this unit exhibits a G+C content (36.4%) similar to that of the remaining of the genome of UWE25 (1931 ORFs, 36.1%). Sixteen homologs to these 28 genes (57%) were found in databases, 12 of them (75%) exhibiting a best score in BLAST analyses with a *Chlamydiaceae *ORF (See additional data file 1). Interestingly, no gene of the other modules of the 100-kb GI exhibited a best hit in similarity analyses with any *Chlamydiaceae *counterpart. Some of the genes present in the first module, such as *sctN *and *sctQ*, are part of a TTSS also present in *Chlamydiaceae*. The other TTSS genes are disseminated along the chromosome of UWE25. The presence of some TTSS genes in the first module and of a gene encoding a putative transposase at the distal end of the first module of this 100-kb GI suggests that this first unit was acquired by chromosomal rearrangements. (See additional data file 1 for the results of BLAST analyses).

Characterized by a low G+C content (34.1%), the 2^nd ^module encodes 18 ORFs. Only five are similar to known protein sequences (28%), four of them being identified as mobility genes (three bacteriophage-related genes and one putative transposase encoding gene).

**Figure 2:**
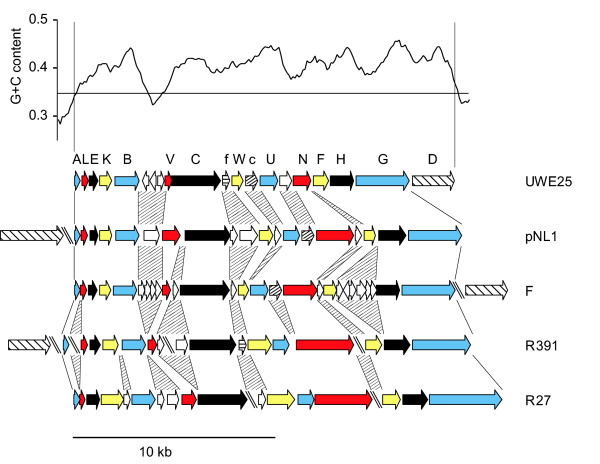
Comparison of the *tra *unit of the endosymbiont UWE25 with similar operons of pNL1 (*Novosphingobium aromaticivorans*), F (*Escherichia coli*), R391 (*Providencia rettgeri*) and R27 (*Salmonella *Typhi) plasmids. In the upper part of the figure: the G+C content of the UWE25 chromosome, around the 1.71 Mb location (1-kb sliding window average, 0.1-kb step). The horizontal line represents the genomic G+C content average. Only the ORFs composed of more than hundred amino-acids are presented on genetic maps of *tra *units/operons by arrows according to their transcription direction (adapted from Lawley *et al*. [20]). Colors or patterns are used to indicate *tra *gene homologs. White genes represent non-conserved transfer genes. Upper case letters refer to the corresponding *tra *genes, whereas lower case letters f and c stand for *trsF *and *trbC*, respectively. Double slashes indicate non-contiguous regions. Interestingly, the G+C-rich genes encoded by the UWE25 chromosome correspond to the ORFs presenting *tra *homologs.

The 3^rd ^module (19 kb), exhibiting the second highest G+C content of the UWE25 genome (40.9%), comprises 21 ORFs. Some of these genes were identified as *tra *genes by Horn *et al*. [14]. Using BLAST analyses and alignment tools, we re-annotated the whole module (see below) and, if we except two transposase genes and one ORF of unknown function, we unveiled that all ORFs of this module belong to a genetic unit similar to the *tra *operons encoding the TFSS previously described in proteobacterial genomes (Figure 2, See additional data file 1 for the re-annotations of this module).

Presenting a low G+C content (33.4%), the 4^th ^module (10 kb) is composed of 13 ORFs. All these ORFs were previously annotated by Horn et al. [14] as encoding hypothetical proteins or without homolog. Our BLAST analysis identified one ORF homologous to genes encoding bacteriophage-related proteins and two genes of proteins involved in DNA metabolism (Table 1 and 2, see also the table in the additional data file 1). Interestingly, a direct repeat is located between the 9th and 10th genes of the module. This 17-bp direct repeat, that presents 3 mismatches is similar to those present at the proximal and distal ends of the GI, exhibiting the same 14 conserved nucleotides. It may reflect a complex evolutionary history of the GI, possibly enabling it to be mobile as 25-kb, 75-kb or 100-kb DNA segments.

A single large protein is encoded along the 5^th ^module (6 kb). Its G+C content is one of the highest of the UWE25 chromosome (41.8%). By BLAST analysis, this protein exhibits the strongest similarity with the human Nod3 protein.

The 6^th ^module (12 kb) is characterized by a low G+C content (33.3%). This unit is composed of 13 ORFs, the first ORF encoding a product similar to the Death on cure (Doc) protein of P1 bacteriophage. Two ORFs code for proteins involved in DNA metabolism and an additional ORF encodes a putative transposase.

The 7^th ^module is short (2 kb) and present a G+C-rich unit (38.7%). Five of the six ORFs of this unit encode a probable resolvase, three putative transposases and a phage-related Doc protein. The final direct repeat is located at the end of this module. With the only exception of the phage-related protein, all other ORFs of the 7^th ^module appear to be similar to gamma-proteobacterial proteins, possibly explaining the observed different signal in the G+C content analysis.

### Role of the type IV secretion system encoded by the 100-kb genomic island

The functions of genes encoded by GIs may be related, among others, to pathogenicity such as the ability to exploit the host intracellular environment. Since no genetic system has been described for any obligate intracellular chlamydiae, we investigated the putative functions of this GI by bioinformatics. We focused our analyses on the TFSS, for which a previous annotation of the *tra *genes showed a genetic unit unable to transfer DNA [14]. Using different protein comparison methods described in the additional data file 1, we identified supplementary *tra *genes, and compared the general organization of this *tra *unit with other genetic elements encoding TFSS genes [20]. The UWE25 *tra *unit displays a striking colinearity with *tra *operons encoding F-like conjugative DNA transfer system, especially to those of the F and pNL1 plasmids of *Escherichia coli *and *Novosphingobium aromaticivorans*, respectively (Figure 2). All homologous genes essential for DNA transfer in plasmid F (*traF*, *traG*, *traH*, *traN*, *traU*, *traW*, and *trbC*) and involved in sex pilus retraction and mating pair stabilization [20] are present, strongly suggesting that, similarly to the other F-like TFSSs, the gene products encoded by the UWE25 *tra *unit are devoted to DNA transfer. With the only exception of *traG*, these genes are not present on P-like and I-like plasmids, reinforcing the close relationship prevailing between the UWE25 *tra *unit and their F-like plasmids counterparts.

**Figure 3:**
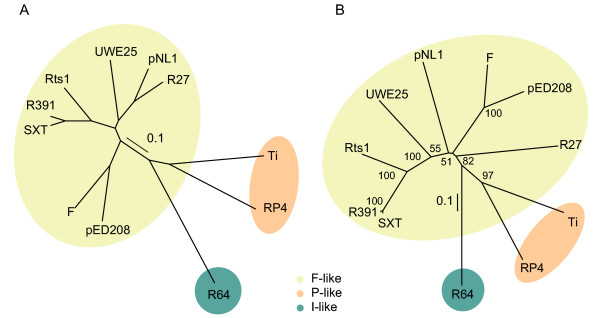
Similarity and phylogenetic analyses of *tra *units showing the close relatedness of the UWE25 *tra *unit with the operons involved in the F-like conjugative systems: **(A) **UPGMA tree of gene order analysis and **(B) **UPGMA tree comparing the Kimura corrected *p*-distances of the concatenated *traA*, *traK*, *traB*, *traV*, and *traC *gene present along the UWE25 *tra *unit and the F-like, I-like and P-like plasmids [20]. The bar represents estimated evolutionary distance scale. The numbers at each node are the results of a bootstrap analysis; each value is derived from 100 samples.

Figure 3 shows that the UWE25 *tra *unit clusters within F-like TFSSs, confirming that it may function as a F-like conjugative system. Drawn as an UPGMA tree (Figure 3A), the comparison of the genetic organization of all *tra *units was performed as a gene order breakpoint analysis developed for the study of the mitochondrial genome evolution [21]. This analysis clearly shows that the closest relatives of the UWE25 *tra *units are the *tra *operons of the F-like conjugative plasmids. The Fitch-Margoliash- and the minimum evolution comparisons performed on the same dataset presented the same tree topologies, confirming the former UPGMA results (data not shown). An *omit *test performed on this tree confirms that the results are robust: with one exception (involving the deep branching of one cluster on one tree), all 11 trees were congruent in all their nodes. Figure 3B shows an UPGMA tree comparing the Kimura corrected *p*-distances (the proportion *p *of nucleotide sites at which two sequences are different, taking into account the proportion of transversion- and transition-substitution rates) of nucleotide sequences of the concatenated *traA*, *traK*, *traB*, *traV*, and *traC *genes. A similar topology is observed with (i) neighbor-joining- and minimum evolution trees inferred using the Kimura-corrected *p*-distances and (ii) UPGMA, neighbor-joining- and minimum evolution trees performed on *p*-distance of the whole coding sequences of the concatenated *tra *genes (See additional data file 2 for these trees). Neighbor-joining- and minimum evolution methods comparing Kimura-corrected *p*-distances of the complete coding sequences confirmed that the *tra *unit of UWE25 is phylogenetically closely related to the *tra *operons of the F-like plasmids: the bootstrap values of 94% and 91% respectively, support the node separating the concatenated *tra *genes of the chromosomal UWE25 and the R27 plasmid, a gamma-proteobacterial F-like conjugative plasmid, from those of all other plasmids (See the additional data file 2 for these trees). In neighbor-joining and minimum evolution analyses of *p*-distances, the *tra *unit of UWE25 also clusters with the *tra *operons of gamma-proteobacterial F-like plasmids: the bootstrap of 96% and 92%, respectively, support the node separating the concatenated *tra *genes of UWE25 and RTS1, SXT, R391, three gamma-proteobacterial F-like conjugative plasmid, from their closest relative, R27 plasmid (See additional data file 2 for these trees). Taken together, all these data strongly suggest that the UWE25 *tra *unit is closely related to F-like conjugative *tra *operons.

### Origin of the genomic island and of its type four secretion system

Our BLAST analyses [22] reveal that a majority (24/43) of genes not presenting a best hit for chlamydial genes but having homologs in other taxa are more related to proteobacterial genes (see Table 2 and the additional data file 1 for similarity analyses indicating for each parachlamydial *tra *gene the most similar gene and its taxonomical background). Moreover, the BLAST analyses of the 21 ORFs of the third module, encoding the *tra *genes, show that most ORFs of this unit (15/21) are of proteobacterial origin. However, since six of them present the highest similarity to alpha-proteobacterial genes and six others to gamma-proteobacterial genes, a more precise origin of the parachlamydial *tra *unit could not be precisely defined by this first approach.

The presence of *gly-tRNA *at the proximal end of the GI of UWE25 is consistent with a close relatedness between this GI and proteobacteria: out of 14 GIs described in the *Islander *database of Mantri *et al*. [15,22] inserted along a chromosome by a *gly-tRNA *(14/140), 12 of them (86%) were sequenced in a proteobacterial genome. No GI of Gram-positives described in the Islander database are inserted in a chromosome within a *gly-tRNA *gene. Again, a precise proteobacterial origin could not be proposed, because the distribution of *gly-tRNA *genes in alpha- (4/22) and gamma-proteobacterial (8/72) GIs is not significantly different: by including only the non-redundant GIs, the distribution of *gly-tRNA *genes in alpha and gamma-proteobacterial GIs is 2/20 and 7/71, respectively.

Comparison of gene order between all *tra *units also failed in assigning a precise origin to the UWE25 *tra *unit since it branched near the alpha- and gamma-proteobacterial *tra *operons (Figure 3A). The only first hint of a possible gamma-proteobacterial origin for the UWE25 *tra *unit was brought by the phylogenetic analyses (Figure 3B and additional files 1 &2). Thus, bootstraps values of 94, 91, 96 and 92% supported the node separating the concatenated *tra *genes of UWE25 and several *tra *operons of gamma-proteobacterial F-like plasmids from the F-plasmids of an alpha-proteobacteria and of other gamma-proteobacteria. (See above, and additional data file 2 for these trees).

## Discussion

We showed that the *Parachlamydia*-related endosymbiont UWE25 presents a 100-kb region largely fullfilling the criteria of GIs [16-18]. Indeed, this DNA region characterized by a high level of gene co-orientation presents a G+C content different from that of the remainder of the genome. The presence of direct repeats flanking this chromosome region enabled us to focus on 100 ORFs. Two identical *gly-tRNA *genes in tandem are present at the proximal end of this genetic element. Moreover, several mobility genes encoding transposases and bacteriophage related-proteins are located within this chromosome region.

The cumulative residual G+C content analysis shows that this GI is composed of seven modules. Such a chimeric organization was already described in other GIs [23,24]. The first module contains chlamydiae genes probably brought by chromosome rearrangements. Some of these genes, homologous to TTSS genes of *Chlamydiaceae*, might provide selective advantages to strains that retained the GI. The 2^nd^, 4^th ^and 6^th ^modules are mainly composed of bacteriophage-related protein genes, that could reflect a putative phage implication in GI formation.

The 3^rd ^module codes for a TFSS similar to *tra *operons. We propose that this *tra *unit is devoted to DNA transfer, based (i) on similarity analyses demonstrating the presence of all genes encoding proteins used during a DNA transfer, (ii) on phylogenetic analyses of *tra *unit genes and, (iii) on comparison of gene order. These analyses clearly demonstrate that the UWE25 *tra *unit is strongly more related to F-like conjugative system than to P-like and I-like secretion systems. The significant bootstraps of all trees obtained by standard gene phylogeny and their congruent topologies with others obtained by the gene order breakpoint analysis not biased by codon usage homing, strongly support the validity of these analyses confirming the F-like conjugative nature of the parachlamydial *tra *unit. Thus, our model significantly differs from the other proposed by Horn *et al*. [14], who did not identify *traA*, *traL*, *traK*, *traV*, and concluded that the UWE25 *tra *unit is involved in protein export, and not in DNA transfer.

The 5^th ^module presents a nucleotide composition similar to the *tra *unit and is composed of a single high G+C 6-kb gene, whose product is similar to the human Nod3 protein. The Nod (Nucleotide-binding oligomerization domain) proteins are members of a family that also includes the apoptosis regulator Apaf1 (Apoptotic protease activating factor 1) and plant disease-resistance gene products [25]. The function of the human Nod3 is still unknown. Like Nod1 and Nod2, Nod3 might be involved in the recognition of conserved motifs present at the surface of bacteria, such as peptidoglycan.

The nucleotide G+C composition of the 2^nd^, 4^th^, and 6^th ^modules are similar, explaining the observed similar negative slope of the residual G+C curves. Moreover, these three modules encode phage-related proteins and proteins involved in DNA metabolism. These modules probably involved in mobility might have a common origin, the ancestral single phage module being currently separated in three pieces by the presence of the *tra *unit and of the Nod3-like protein encoding gene.

The positive slope in the G+C analysis of the 7^th ^module echoes those of the *tra *unit (3^rd ^module) and of the Nod3-like protein (5^th ^module). The 7^th ^module encodes a transposition resolvase and three transposases similar to gamma-proteobacterial homologs. With the only exception of the phage-related Doc protein, that has an homolog at the beginning of the sixth module, and that might be located there after transposition, the 7^th ^module appears thus to have a different origin than the 2^nd^, 4^th ^and 6^th ^modules, though also encoding mobility genes.

The presence of a F-like *tra *unit along the sequences of UWE25 is the first evidence of a putative conjugative system in chlamydiae. If conjugation occurs, it most probably takes place within free-living amoebae, that may contain several hundreds of *Parachlamydia *bacteria tightly packed in their vacuoles [4]. Such a conjugation system would be a mechanism to transfer DNA between internalized bacteria sharing an amoebal vacuole. Moreover, it may provide molecular genetic tools for obligate intracellular bacteria.

The presence of *tra *units/operons in the parachlamydial UWE25 and in proteobacteria could be explained by an emergence of this unit in a common ancestor of both clades, and by its subsequent loss in *Chlamydiaceae*. Another evolutionary scenario is that the *tra *unit was acquired from a proteobacteria by a *Parachlamydiaceae *in a common amoebal vacuole. Since the *tra *unit is absent from all sequenced *Chlamydiaceae *genomes, this transfer would have occurred after the divergence of *Parachlamydiaceae *and *Chlamydiaceae*, at a time when *Parachlamydia *was already an intracellular bacteria. An intra-amoebal transfer of this GI is supported by the permissivity of free-living amoebae to proteobacteria [26], and by several hints suggesting its proteobacterial origin. Though phylogenetic analyses suggested a gamma-proteobacterial origin of the F-like parachlamydial *tra*, further analyses have to confirm whether this GI module was acquired from an alpha-, beta-, or gamma-proteobacteria unit. We hypothesize that the F-like parachlamydial *tra *unit has been brought by a lateral transfer from a proteobacterial genome. This hypothesis is strongly supported by the cumulative GC skew analysis [27-30] producing a signal of the GI differing from that of the remaining of the genome (Figure 1A and 1B). The value of nucleotide skew analyses as good taxonomical markers is supported by (i) routine analyses on prokaryotic genome by cumulative TA-skews [30] and (ii) comparison of intragenic nucleotide skews of small subunit ribosomal RNA of the whole living world [31]. The genometric approach appeared to be able to identify GIs of *Chlamydiales*. Sequencing additional genomes of environmental chlamydiae, that present a large biodiversity [3], will provide major insights on bacterial evolution and hopefully a better comprehension of the emergence of this parachlamydial GI.

## Conclusions

We showed that a GI present on the UWE25 chromosome encodes a potentially functional F-like DNA conjugative system. This is the first hint of a putative conjugative system in chlamydiae. Conjugation most probably occurs within free-living amoebae, that may contain hundreds of *Parachlamydiaceae *bacteria tightly packed in vacuoles. Such a conjugative system might be involved in DNA transfer between internalized bacteria. Since this system is absent from the sequenced genomes of *Chlamydiaceae*, we hypothesize that it was acquired after the divergence between *Parachlamydiaceae *and *Chlamydiaceae*, when the *Parachlamydia*-related symbiont was an intracellular bacteria. It suggests that this heterologous DNA was acquired by a *Parachlamydiaceae *from phylogenetically-distant bacteria sharing an amoebal vacuole. Since *Parachlamydiaceae *are emerging agents of pneumonia [2] and since many GIs are also considered as pathogenicity islands [17], the Pam100G GI might be involved in pathogenicity. In future, conjugative systems might be developed as genetic tools for studying *Chlamydiales*.

## Methods

### Sequence

The genome sequence of UWE25 [14] (Accession number: NC_005861) is available at the NCBI website [32,33]. In this contribution, the acronym UWE25 refers only to the *Parachlamydia*-related endosymbiont UWE25, and thus not to the *Acanthamoeba *sp. strain UWE25 from which the parachlamydial endosymbiont UWE25 was recovered [3]. Horn *et al*. recently proposed UWE25 as the type strain of a new bacterial species: *Protochlamydia amoebophila *[34].

### BLAST analyses

BLAST analyses were performed with BLASTP 2.2.9 [35] available on the NCBI website [36] using the BLOSUM62 matrix, and gap penalties of 11 and 1. Each ORF was compared against all genes of non-redundant databases available at the NCBI website. An e-value of 0.001 was selected as a standard cut-off. To further identify possible homologous ORFs, we also BLASTed each *tra *gene of F plasmid versus all genes of the full genome of *Parachlamydia *and conversely, each ORF of the putative parachlamydial *tra *unit versus counterparts of the different F-like plasmids. CLUSTALW was used to detect the best relatedness of a given parachlamydial Tra protein with its possible homologs encoded by the F and pNL1 plasmids.

### Residual cumulative GC content

The residual cumulative G+C content, a slightly modified version of the cumulative GC profile defined by Zhang and Zhang [19], was used to reveal local variations of G+C content of a genome, without using sliding windows of arbitrary size. First, a G+C content analysis was performed on 100-bp windows of the selected chromosome sequence, as for a cumulative GC skew analysis. The cumulative G+C content *GC*_*n *_of the *n*^*th *^window is obtained by cumulating the G+C contents from the first to the *n*^*th *^window:


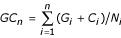


where, in the window *i*, *G*_*i *_and *C*_*i *_are the numbers of Gs and Cs, respectively, and *N*_*i *_is the total number of nucleotides. To visualize genomic regions differing from the average G+C content, a linear regression *y *defined by a slope *k *is performed on the cumulative curve using the least square methods:

*y*(*n*) = *kn*

where *n *is the position of the center of the *n*^th ^window. The residual cumulative G+C content curve *GC*' can then be drawn as a function of the position of each window center:

*GC*'_*n *_= *GC*_*n *_- *kn*

Zhang and Zhang [19] recently demonstrated that, in some instances, abrupt changes in the residual cumulative G+C content curve correspond to genomic islands.

### Repeats identification

The perfect tandem repeats identification was first performed using the EQUICKTANDEM software (Richard Durbin, Sanger Institute, Wellcome Trust Genome Campus, Hinxton, Cambridge, CB10 1SA, UK) [37] on a 200-kb DNA sequence (UWE25 genome position: 1.6 to 1.8 Mb) encompassing the *tra *genes previously identified by Horn *et al*. [14]. The duplicated genes and the ORFs containing internal repeats were removed. For each pair of direct repeats, potential unperfect matches of flanking nucleotides were scanned using DNA strider 1.2.1 [38], with the following settings: a minimal size of 11 bp and 3 mismatches. Furthermore, sequences similar to direct repeats were searched along the whole chromosome, and sequences also found outside the selected 200-kb region were discarded from our analysis. Finally, the direct repeats positions were compared to the G+C content analysis, the cumulative GC skew curve, and to tRNA genes locations.

### Phylogenetic analyses

Since Horn *et al*. [14] did not identified *traA*, *traL*, *traK*, *traV*, re-annotation of the UWE25 *tra *unit was necessary for phylogenetic analyses. We used i) the genes of F-like plasmids encoding the following *tra *genes, i.e. *traA*, *traK*, *traB*, *traV*, *traC*, and ii) the corresponding ORFs of P- and I-like plasmids [20], i.e. *trbC/VirB2*, *trbG/VirB9*, *trbI/VirB10*, *trbH/VirB7*, *trbE/VirB4 *of P-plasmids and *traX*, *traN*, *traO*, *traI*, *traU *of I-plasmids, respectively. The genes were concatenated to obtain a single nucleotide sequence and aligned with CLUSTALW ([39] as it was already performed for genes of ribosomal proteins [40]. Using this alignment and the MEGA 2.1 software [41], we inferred phylogenetic relationships by drawing trees using *p*-distances (the proportion *p *of nucleotide sites at which two sequences compared are different) and Kimura corrected *p*-distance (correction for the rates of transition and transversion) with Unweighted Pair Group Method with Arithmetic Mean (UPGMA), neighbor-joining, and minimum evolution methods. To prevent alignment biases, trees were drawn using the complete deletion option implemented on MEGA 2.1.

### Gene order breakpoint analyses

To quantify the inversion and transposition events leading to the current organization of *tra *operons, the gene order breakpoint analysis developed for small genomes (mitochondria) by Blanchette *et al*. [21] was used to estimate the similarity of gene order existing between the *tra *unit of UWE25 and the *tra *operons reviewed by Lawley *et al*. [20]. The distance calculated for two given operons *O*_*i *_and *O*_*j *_containing homologous genes proposed by Blanchette *et al*. [21] was slightly modified to take into account the variation of gene numbers of *tra *operons: instead of counting the number of minimal breakpoints existing between two *tra *operons, a distance was estimated by measuring the proportion of conserved gene pairs between both genomic entities. Next, a comparison matrix is established by calculating the distance for each pairwise comparison. Finally, a dissimilarity matrix is obtained by subtracting each distance from 1.

For instance, if the operon *O*_*i *_encodes sequentially four genes (*a*, *b*, *c*, and *d*) and the operon *O*_*j*_, six genes (*a*, *b*, *e*, *-d*, *-c*, and *-f*; genes labeled by a minus sign are encoded by the complementary strand), the gene order breakpoint analysis reveals that two gene pairs are conserved: *ab *and *cd*. The dissimilarity distances existing between the operons i) *O*_*i *_and *O*_*j*_, and ii) *O*_*j *_and *O*_*i *_would be: 1-(2/3) = 1/3 and 1-(2/5) = 3/5, respectively.

From the square dissimilarity matrix, phylogenetic trees were drawn. Three different distance-matrix analyses were used: the UPGMA, the Fitch-Margoliash- and the minimum evolution methods. To assess the robustness of the tree, an *omit *test [42] was performed on 11 UPGMA trees, in each one organism is missing.

## Authors' contributions

GG and CAR initiated the project. GG and FC reannotated the parachlamydial *tra *unit and performed all BLAST analyses. FC and LG delimited the GI by direct repeat analyses. GG drew the phylogenetic trees of the concatenated *tra *genes. After developing the residual cumulative G+C content analysis used for a software development, LG performed the G+C content analyses and the gene order comparison of *tra *units by the gene order breakpoint analysis. CAR established the correlation existing between the tRNA genes and the GIs according to taxonomy and coordinated the team work. GG wrote the first draft of the paper. All authors improved the manuscript and approved its final version.

## Supplementary Material

Additional File 1 Supplementary table. Results of BLAST [35,36] analyses of 100 ORFs present in the 100-kb region. BLAST analyses were performed using BLOSUM62 matrix and gap penalties of 11 and 1. Chromosome location of each ORF, its G+C content, coding strand, and the presence of at least one homolog in *Chlamydiaceae *are presented. Direct repeats (DR), *gly-tRNA *genes and limits of each modules of the GI are highlighted. Click here for file

Additional File 2 Supplementary figure. Phylogenetic analyses suggest that the UWE25 *tra *unit is phylogenetically closely related to F-like DNA conjugative *tra *operons: (A) UPGMA-, (B) Neighbor-joining-, and (C) minimum evolution-trees comparing p-distances and Kimura corrected *p*-distances of nucleotide sequences of the concatenated *traA*, *traK*, *traB*, *traV*, and *traC *genes (the UPGMA tree comparing the Kimura corrected *p*-distances of *tra *genes, shown in Figure 3B, is presented here to facilitate comparison with the other trees). Interestingly, in neighbor-joining and minimum evolution analyses of the *p*-distances, the *tra *unit of UWE25 is clustered with *tra *operons of gamma-proteobacterial F-like plasmids: bootstrap values of 96% and 92%, respectively, support the node separating the concatenated *tra *genes of UWE25 and RTS1, SXT, R391, three gamma-proteobacterial F-like conjugative plasmids, from their closest relative R27 plasmid. Similarly, in neighbor-joining and minimum evolution analyses of the Kimura corrected *p*-distances, bootstrap values of 94% and 91%, respectively, support the node separating the concatenated *tra *genes of the chromosomal UWE25 and the R27 plasmid, another gamma-proteobacterial F-like conjugative plasmid, from those of all other plasmids. Click here for file

## Figures and Tables

**Table 1:** Description of main features of the parachlamydial 100-kb genomic island. Chromosome location of direct repeats, tRNA genes, *tra *operon, transposases, bacteriophage-related proteins and proteins involved in DNA metabolism is listed below.

	Protein number^a^	Position^a^
Direct repeat	-	1648147–1648157
*gly-tRNA*^a,b^	-	1648172–1648243
*gly-tRNA*^a,b^	-	1648332–1648403
Transposase^c^	pc1402	1679924–1680400
Phage-related protein^c^	pc1404	1681569–1682441
Putative transcriptional regulator^c,d^	pc1405	1679924–1680400
Phage-related protein^c^	pc1410	1685329–1686447
Putative transposase^c^	pc1419	1695418–1696245
*tra *operon^e^	pc1420-1441	1696410–1716241
Transposases^e^	pc1426-1427	1700887–1701896
Putative DNA-binding protein^c^	pc1443	1716648–1717004
Phage-related protein^c^	pc1444	1717137–1717400
Direct repeat	-	1723093–1723103
Putative ATPase involved in DNA repair^c^	pc1451	1723169–1723504
Probable Doc (death on cure) protein, bacteriophage P1^a^	pc1456	1732622–1732999
Putative DNA-binding protein	pc1461	1735745–1736065
Putative transposase^c^	pc1465	1740371–1741198
Probable DNA double-strand break repair ATPase	pc1467	1742079–1744181
Putative transposase	pc1468	1744634–1745023
Probable resolvase^a^	pc1469	1745398–1745955
Probable transposases, partial length^a^	pc1470-1471	1745807–1746692
Probable Doc (death on cure) protein, bacteriophage P1^a^	pc1473	1747135–1747512
Phage-related protein^c^	pc1474	1747618–1747809
Direct repeat	-	1747915–1747925

^a^, according to Horn *et al*. [14];

^b^, positive strand (cooriented as the majority of the genes of the GI); *gly-tRNA*s are separated by 88 nt;

^c^, identified by BLAST [35; 36] and CLUSTALW [39] by ourselves;

^d^, phage-related protein based on additional BLAST hit;

^e^, partially annotated by Horn *et al*. [14] and further characterized by ourselves by BLAST [35, 36].

**Table 2:** The genomic island of the UWE25 endosymbiont presents seven different modules. The limit of each module was determined by residual cumulative G+C content analysis.

Modules	1^st ^mod.	2^nd ^mod.	3^rd ^mod.	4^th ^mod.	5^th ^mod.	6^th ^mod.	7^th ^mod.
Length	32 kb	16 kb	19 kb	10 kb	6 kb	12 kb	2 kb
Mean G+C content (%)	36.4%^a^	34.1%	40.9%	33.4%	41.8%	33.3%	38.7%
Number of ORFs	28	18	21	13	1	13	6
							
Number of genes having an homolog	16 (57%)	5 (28%)	16 (85%)	7 (54%)	1 (100%)	5 (38%)	5 (83%)
Number of genes having no homolog	12	13	5	6	0	8	1
							
Best hits with homologs in:							
							
- chlamydiae	12	0	0	0	0	0	0
- cyanobacteria	2	2	0	3	0	2	0
- plants^b^	0	0	0	0	0	0	0
- α-proteobacteria	0	0	6	0	0	0	0
- β-proteobacteria	1	0	3	1	0	0	0
- γ-proteobacteria	0	1	6	2	0	0	4^c^
- Bacteroidetes group	0	0	1	0	0	2	1
- others	1	2	0	1	1	1	0
							
Homologous^d ^to:							
- phage-related protein	0	3	0	1	0	1	2
- putative transposase	1	1	2	0	0	2^e^	2
- resolvase	0	0	0	0	0	0	1
- protein involved in DNA metabolism	0	1	0	2	0	2	0

^a ^the G+C content is similar to that of 36.1% of the remaining genome (calculated on 1931 ORFs);

^b ^5% of the 2031 ORFs of the genome of UWE25 have products homologous to plant proteins, but no ORFs of the GI were homologous to plant counterparts;

^c ^two ORFs which presented best homologs encoded by a plasmid of an uncultured bacteria present in activated sludge have a second best BLAST hit encoded by a gamma-proteobacterial ORFs (*Pseudomonas *sp.);

^d ^not only the best BLAST hit is taking into consideration to determine the putative function encoded by the ORF;

^e ^one of them has an e-value above 0.001.
