# Supplementary material for: A genomic island present along the bacterial chromosome of the Parachlamydiaceae UWE25, an obligate amoebal endosymbiont, encodes a potentially functional F-like conjugative DNA transfer system
Source: BMC Microbiol. 2004 Dec 22;4:48. doi: 10.1186/1471-2180-4-48 (PMC548262; doi:10.1186/1471-2180-4-48)
Supplement: Additional File 1 — Supplementary table. Results of BLAST [35,36] analyses of 100 ORFs present in the 100-kb region. BLAST analyses were performed using BLOSUM62 matrix and gap penalties of 11 and 1. Chromosome location of each ORF, its G+C content, coding strand, and the presence of at least one homolog in Chlamydiaceae are presented. Direct repeats (DR), gly-tRNA genes and limits of each modules of the GI are highlighted. [file 1471-2180-4-48-S1.doc]

**Supplementary table**. Results of BLAST [35, 36] analyses of 100 ORFs present in the 100-kb region. BLAST analyses were performed using BLOSUM62 matrix and gap penalties of 11 and 1. Chromosome location of each ORF, its G+C content, coding strand, and the presence of at least one homolog in *Chlamydiaceae* are presented. Direct repeats (DR), *gly-tRNA* genes and limits of each modules of the GI are highlighted in grey.

|  | |  |  |  |  | BLAST results | | | | | | |
| --- | --- | --- | --- | --- | --- | --- | --- | --- | --- | --- | --- | --- |
| Location^a^ | | ORFs number^a^ | G + C content | Strand^a^ | Homolog(s) in  Chlamydiaceae^a^ | Score  (bits) | *E*-value  (log) | Similarity (%) | Identity (%) | Best hit | Species | Taxon^b^ |
| 1648147 | 1648157 | 1^st^ DR |  |  |  |  |  |  |  |  |  |  |
| 1648172 | 1648243 | *gly-tRNA* 1 |  | + |  |  |  |  |  |  |  |  |
| 1648332 | 1648403 | *gly-tRNA* 2 |  | + |  |  |  |  |  |  |  |  |
| 1648434 | 1649741 | pc1375 | 0.371 | + | Yes | 141 | -32 | 50 | 27 | Trigger factor-peptidyl-prolyl isomerase | *Chlamydophila pneumoniae* | Ch |
| 1650179 | 1650802 | pc1376 | 0.389 | + | Yes | 289 | -77 | 86 | 71 | ATP-dependent Clp protease proteolytic subunit 2 | *Chlamydia muridarum* | Ch |
| 1651041 | 1652282 | pc1377 | 0.357 | + | Yes | 513 | -144 | 76 | 66 | ATP-dependent Clp protease ATP-binding subunit ClpX | *Chlamydia trachomatis* | Ch |
| 1652477 | 1653226 | pc1378 | 0.363 | + | No | 99 | -19 | 48 | 27 | Histone acetylTransferase HPA2 and related acetylTransferases | *Nostoc punctiforme* | Cy |
| 1654154 | 1653231 | pc1379 | 0.314 | - | No | 103 | -20 | 50 | 32 | Histone acetylTransferase HPA2 and related acetylTransferases | *Trichodesmium erythraeum* | Cy |
| 1654345 | 1656954 | pc1380 | 0.353 | + | No |  |  |  |  | No hits |  |  |
| 1657029 | 1658273 | pc1381 | 0.369 | + | No |  |  |  |  | No hits |  |  |
| 1658367 | 1659815 | pc1382 | 0.374 | + | No |  |  |  |  | No hits |  |  |
| 1659904 | 1661211 | pc1383 | 0.355 | + | No |  |  |  |  | No hits |  |  |
| 1661342 | 1661914 | pc1384 | 0.342 | + | Yes | 95 | -18 | 57 | 34 | Type III secretion chaperone SycD | *Chlamydophila caviae* | Ch |
| 1662033 | 1664282 | pc1385 | 0.391 | + | No |  |  |  |  | No hits |  |  |
| 1664295 | 1664888 | pc1386 | 0.325 | + | Yes | 61 | -8 | 46 | 26 | Low calcium response protein H | *Chlamydophila pneumoniae* | Ch |
| 1664891 | 1665565 | pc1387 | 0.378 | + | No |  |  |  |  | No hits |  |  |
| 1665596 | 1666546 | pc1388 | 0.322 | + | No |  |  |  |  | No hits |  |  |
| 1666578 | 1667660 | pc1389 | 0.367 | + | No |  |  |  |  | No hits |  |  |
| 1667910 | 1668296 | pc1390 | 0.333 | + | Yes | 49 | -4 | 53 | 22 | Conserved hypothetical protein | *Chlamydophila caviae* | Ch |
| 1668394 | 1670223 | pc1391 | 0.363 | + | Yes | 338 | -91 | 57 | 38 | Fha domain (homolog to adenylate cyclase) | *Chlamydophila pneumoniae* | Ch |
| 1670270 | 1670512 | pc1392 | 0.288 | + | Yes |  |  |  |  | No hits |  |  |
| 1670600 | 1671064 | pc1393 | 0.333 | + | Yes | 80 | -14 | 65 | 36 | Conserved hypothetical protein | *Chlamydia trachomatis* | Ch |
| 1671073 | 1671756 | pc1394 | 0.330 | + | No | 145 | -33 | 54 | 37 | *N*-Acetylglucosamide-(beta 1-4)-galactosyl-transferase; lactose synthase | *Homo sapiens* | Eu |
| 1672066 | 1672344 | pc1395 | 0.398 | + | Yes |  |  |  |  | No hits |  |  |
| 1672490 | 1673341 | pc1396 | 0.363 | + | No |  |  |  |  | No hits |  |  |
| 1673399 | 1674763 | pc1397 | 0.406 | + | Yes | 604 | -171 | 86 | 71 | Type III secretion cytoplasmic ATPase SctN (YopN) | *Chlamydophila pneumoniae* | Ch |
| 1674777 | 1675274 | pc1398 | 0.333 | + | Yes | 64 | -9 | 60 | 43 | Conserved hypothetical protein | *Chlamydia trachomatis* | Ch |
| 1675345 | 1676337 | pc1399 | 0.388 | + | No |  |  |  |  | No hits |  |  |
| 1676642 | 1677979 | pc1400 | 0.374 | + | Yes | 63 | -8 | 54 | 35 | Type III secretion Translocase SctQ | *Chlamydophila caviae* | Ch |
| 1678055 | 1679644 | pc1401 | 0.369 | + | Yes | 230 | -59 | 60 | 42 | Serine/threonine-protein kinase | *Chlamydophila caviae* | Ch |
| 1679924 | 1680400 | pc1402 | 0.440 | + | No | 228 | -58 | 88 | 76 | Possible transposase | *Nitrosomonas europaea* | β |
| 1680650 |  |  |  |  |  |  |  |  |  |  |  |  |
| 1681563 | 1681162 | pc1403 | 0.303 | - | Yes |  |  |  |  | No hits |  |  |
| 1682441 | 1681569 | pc1404 | 0.353 | - | No | 112 | -23 | 61 | 46 | Conserved hypothetical protein^c^ | *Thermoanaerobacter tengcongensis* | Fi |
| 1683099 | 1682779 | pc1405 | 0.305 | - | Yes | 52 | -5 | 59 | 35 | Predicted transcriptional regulator^d^ | *Pseudomonas aeruginosa* | γ |
| 1683529 | 1683825 | pc1406 | 0.323 | + | Yes |  |  |  |  | No hits |  |  |
| 1683880 | 1684227 | pc1407 | 0.359 | + | Yes |  |  |  |  | No hits |  |  |
| 1684227 | 1684778 | pc1408 | 0.342 | + | No |  |  |  |  | No hits |  |  |
| 1685087 | 1684827 | pc1409 | 0.314 | - | Yes |  |  |  |  | No hits |  |  |
| 1686447 | 1685329 | pc1410 | 0.377 | - | No | 360 | -98 | 71 | 51 | Unknown protein ^e^ | *Anabaena variabilis* | Cy |
| 1686882 | 1687082 | pc1411 | 0.388 | + | Yes |  |  |  |  | No hits |  |  |
| 1687470 | 1687673 | pc1412 | 0.324 | + | No |  |  |  |  | No hits |  |  |
| 1688222 | 1687923 | pc1413 | 0.373 | - | Yes | 58 | -7 | 56 | 33 | Hypothetical protein | *Treponema denticola* | Sp |
| 1688523 | 1688209 | pc1414 | 0.324 | - | No |  |  |  |  | No hits |  |  |
| 1689469 | 1690965 | pc1415 | 0.335 | + | No |  |  |  |  | No hits |  |  |
| 1691344 | 1691529 | pc1416 | 0.339 | + | No |  |  |  |  | No hits |  |  |
| 1691799 | 1693271 | pc1417 | 0.314 | + | No |  |  |  |  | No hits |  |  |
| 1693840 | 1694943 | pc1418 | 0.385 | + | No |  |  |  |  | No hits |  |  |
| 1695418 | 1696245 | pc1419 | 0.325 | + | No | 168 | -40 | 55 | 34 | Hypothetical protein ^f^ | *Nostoc punctiforme* | Cy |
| 1696814 | 1696410 | pc1420 | 0.311 | - | Yes |  |  |  |  | No hits |  |  |
| 1697150 |  |  |  |  |  |  |  |  |  |  |  |  |
| 1697418 | 1697735 | pc1421 | 0.390 | + | Yes^g^ |  |  |  |  | TraA^h^ | Not determined ^h^ |  |
| 1697757 | 1698062 | pc1422 | 0.359 | + | Yes^g^ | 32 | 2 | 46 | 31 | TraL - plasmid pNL1^i, j^ | *Novosphingobium aromaticivorans* | α |
| 1698071 | 1698640 | pc1423 | 0.395 | + | No | 75 | -12 | 47 | 31 | Conserved hypothetical protein (TraE)^k^ | *Dechloromonas aromatica* | β |
| 1698641 | 1699333 | pc1424 | 0.408 | + | No | 35 | 1 | 42 | 21 | Conjugative transfer protein TraK ^h, j^ | *Vibrio vulnificus*^j^ | γ |
| 1699404 | 1700630 | pc1425 | 0.425 | + | No | 133 | -30 | 57 | 39 | Dehydrogenase – (TraB)^l^ | *Dechloromonas aromatica* | β |
| 1701381 | 1700887 | pc1426 | 0.331 | - | No | 62 | -8 | 49 | 35 | Tn*10*-like transposases | *Bacteroides uniformis* | Ba |
| 1701896 | 1701456 | pc1427 | 0.313 | - | Yes | 78 | -13 | 55 | 34 | Putative transposase | *Acinetobacter baumannii* | γ |
| 1701958 | 1702431 | pc1428 | 0.399 | + | No |  |  |  |  | No hits |  |  |
| 1702424 | 1702798 | pc1429 | 0.403 | + | Yes^g^ |  |  |  |  | TraV^m^ | Not determined ^i^ |  |
| 1702800 | 1705286 | pc1430 | 0.408 | + | No | 325 | -87 | 43 | 27 | TraC - plasmid pNL1 | *Novosphingobium aromaticivorans* | α |
| 1705274 | 1705756 | pc1431 | 0.408 | + | No | 72 | -11 | 57 | 38 | Protease TraF (TrsF)^n^ | *Dechloromonas aromatica* | β |
| 1705741 | 1706373 | pc1432 | 0.406 | + | No | 119 | -25 | 55 | 37 | TraW | *Novosphingobium aromaticivorans* | α |
| 1706382 | 1706978 | pc1433 | 0.424 | + | No | 73 | -11 | 47 | 29 | Conjugative transfer protein TrbC - plasmid F | *Escherichia coli* | γ |
| 1707047 | 1707982 | pc1434 | 0.441 | + | No | 243 | -63 | 59 | 40 | TraU - plasmid pNL1 | *Novosphingobium aromaticivorans* | α |
| 1707925 | 1708692 | pc1435 | 0.379 | + | No |  |  |  |  | No hits |  |  |
| 1708784 | 1708969 | pc1436 | 0.409 | + | No |  |  |  |  | No hits |  |  |
| 1709083 | 1709637 | pc1437 | 0.422 | + | No | 100 | -20 | 57 | 39 | TraN - plasmid pNL1 | *Novosphingobium aromaticivorans* | α |
| 1709634 | 1710425 | pc1438 | 0.404 | + | No | 94 | -18 | 42 | 27 | TraF ^n^ | *Escherichia coli* | γ |
| 1710422 | 1711780 | pc1439 | 0.422 | + | No | 196 | -49 | 55 | 31 | TraH protein precursor | *Salmonella* Typhimurium | γ |
| 1711791 | 1714574 | pc1440 | 0.422 | + | No | 140 | -32 | 41 | 24 | Large exoproteins involved in heme utilization or adhesion (TraG)^o^ | *Rhodobacter sphaeroides* | α |
| 1714571 | 1716241 | pc1441 | 0.429 | + | No | 278 | -73 | 51 | 31 | Membrane protein TraD - plasmid R100 | *Escherichia coli* | γ |
| 1716150 |  |  |  |  |  |  |  |  |  |  |  |  |
| 1716302 | 1716655 | pc1442 | 0.331 | + | Yes | 71 | -11 | 60 | 41 | Conserved hypothetical protein | *Shewanella oneidensis* | γ |
| 1716648 | 1717004 | pc1443 | 0.350 | + | Yes | 63 | -8 | 54 | 36 | Putative DNA-binding protein | *Bordetella parapertussis* | β |
| 1717137 | 1717400 | pc1444 | 0.314 | + | Yes |  |  |  |  | No hits ^p^ |  |  |
| 1717579 | 1717860 | pc1445 | 0.365 | + | No | 49 | -4 | 63 | 39 | Conserved hypothetical protein | *Anabaena variabilis* | Cy |
| 1718022 | 1718702 | pc1446 | 0.373 | + | No | 258 | -67 | 76 | 56 | Conserved hypothetical protein | *Anabaena variabilis* | Cy |
| 1718994 | 1719326 | pc1447 | 0.351 | + | Yes |  |  |  |  | No hits |  |  |
| 1719350 | 1720123 | pc1448 | 0.320 | + | No |  |  |  |  | No hits |  |  |
| 1722591 | 1720498 | pc1449 | 0.330 | - | No | 69 | -10 | 42 | 28 | Conserved hypothetical protein | *Gibberella zeae* | Eu |
| 1722979 | 1722785 | pc1450 | 0.323 | - | Yes |  |  |  |  | No hits |  |  |
| 1723093 | 1723103 | 2^nd^ DR ^q^ |  |  |  |  |  |  |  |  |  |  |
| 1723169 | 1723504 | pc1451 | 0.372 | - | Yes | 57 | -7 | 70 | 32 | ATPase involved in DNA repair | *Actinobacillus pleuropneumoniae* | γ |
| 1723987 | 1725021 | pc1452 | 0.302 | + | No |  |  |  |  | No hits |  |  |
| 1725568 | 1725891 | pc1453 | 0.358 | + | No | 122 | -27 | 75 | 56 | HicB protein | *Nostoc* sp. | Cy |
| 1726124 | 1726315 | pc1454 | 0.302 | + | No |  |  |  |  | No hits |  |  |
| 1726250 |  |  |  |  |  |  |  |  |  |  |  |  |
| 1726283 | 1731883 | pc1455 | 0.417 | - | No | 286 | -77 | 55 | 42 | NOD3 protein ^r^ | *Homo sapiens* | Eu |
| 1731450 |  |  |  |  |  |  |  |  |  |  |  |  |
| 1732622 | 1732999 | pc1456 | 0.328 | - | No | 111 | -24 | 62 | 44 | Death on cure protein ^s^ | *Chlorobium tepidum* | Ba |
| 1732996 | 1733226 | pc1457 | 0.311 | - | No | 46 | -4 | 51 | 36 | Hypothetical protein | *Chlorobium tepidum* | Ba |
| 1733583 | 1733768 | pc1458 | 0.327 | - | No |  |  |  |  | No hits |  |  |
| 1734037 | 1734762 | pc1459 | 0.348 | - | No |  |  |  |  | No hits |  |  |
| 1734915 | 1735115 | pc1460 | 0.333 | - | No |  |  |  |  | No hits |  |  |
| 1735745 | 1736065 | pc1461 | 0.336 | - | Yes | 56 | -7 | 61 | 44 | Hypothetical protein^t^ | *Gloeobacter violaceus* | Cy |
| 1736478 | 1739090 | pc1462 | 0.352 | + | No |  |  |  |  | No hits |  |  |
| 1739087 | 1739275 | pc1463 | 0.291 | - | No |  |  |  |  | No hits |  |  |
| 1739816 | 1740037 | pc1464 | 0.387 | + | No |  |  |  |  | No hits |  |  |
| 1740371 | 1741198 | pc1465 | 0.327 | - | No | 168 | -41 | 55 | 34 | Hypothetical protein^u^ | *Nostoc punctiforme* | Cy |
| 1741400 | 1742194 | pc1466 | 0.311 | - | No |  |  |  |  | No hits |  |  |
| 1742079 | 1744181 | pc1467 | 0.305 | - | No | 73 | -12 | 43 | 22 | Probable DNA double-strand break repair ATPase | *Aquifex aeolicus* | Aq |
| 1744634 | 1745023 | pc1468 | 0.382 | + | Yes |  |  |  |  | No hits^v^ |  |  |
| 1745350 |  |  |  |  |  |  |  |  |  |  |  |  |
| 1745398 | 1745955 | pc1469 | 0.441 | + | No | 285 | -76 | 88 | 79 | Transposition resolvase | *Escherichia coli* | γ |
| 1745807 | 1746337 | pc1470 | 0.382 | + | No | 173 | -42 | 81 | 69 | Transposase | Uncultured bacteria ^w^ | - |
| 1746372 | 1746692 | pc1471 | 0.439 | + | No | 147 | -35 | 81 | 68 | Transposase | Uncultured bacteria ^w^ | - |
| 1746893 | 1747138 | pc1472 | 0.317 | + | No |  |  |  |  | No hits |  |  |
| 1747135 | 1747512 | pc1473 | 0.354 | + | Yes | 69 | -11 | 45 | 29 | Death on cure protein^s^ | *Chlorobium tepidum* | Ba |
| 1747618 | 1747809 | pc1474 | 0.312 | + | No | 45 | -4 | 78 | 75 | Hypothetical phage-related protein | *Pseudomonas* sp*.* | γ |
| 1747915 | 1747925 | 3^rd^ DR |  |  |  |  |  |  |  |  |  |  |

^a^, according to Horn *et al.* [14];

^b^, stand for: Ch, *Chlamydiales*; Cy, cyanobacteria; α, β, and γ,  alpha-, beta- and gamma-proteobacteria, respectively; Eu, eucaryotes; Ba, bacteroidetes group; Fi, firmicutes; Sp, spirochaetes; Aq, aquificae;

^c^, phage-related protein based on additional BLAST hits: this ORF encodes an homolog of the DnaD replication protein of prophage lamda (score of 91, e-value of 9x10 ^-17^, 58% similarity, 37% identity);

^d^, phage-related protein based on additional BLAST hits (score of 48, e-value of 5x10 ^-4^, 59% similarity, 40% identity);

^e^, phage-related protein based on additional BLAST hits (score of 342, e-value of 9x10 ^-92^, 72% similarity, 50% identity);

^f^, putative transposase based on additional BLAST hits: this ORF encodes an homolog of a transposase of *Enterococcus faecium* (score of 73, e-value of 9x10 ^-11^, 49% similarity, 29% identity);

^g^, Horn *et al.* [14] found genes homologous to these *tra* genes in the genome of *Chlamydiaceae;* if this is confirmed, it will suggest that the parachlamydial *tra* unit was already present in the common ancestor of both *Chlamydiaceae* and *Parachlamydiaceae.* However, by BLAST analyses using an *E*-value threshold of 0.001, we find only short homologous sequences of 15 to 18 nucleotides in *Chlamydiaceae*;

^h^, identified using CLUSTALW [39] comparisons between the UWE25 *tra* unit- and the other F-like system sequences previously reported by Lawley *et al.* [20];

^i^, identified by BLAST comparisons performed on the F-like system sequences previously reported by Lawley *et al.* [20];

^j^, identified by BLAST using a *E*-value cut-off of 0.001;

^k^, annotated as putative TraE by Horn *et al.* [14], BLASTs also with TraE of plasmid pNL1 of *N. aromaticivorans*;

^l^, annotated as putative TraB by Horn *et al.* [14], BLASTs also with TraB of plasmid pSLT of *S. enterica* serovar Typhimurium;

^m^, identified using the colinearity analyses performed on the F-like system sequences previously rewieved by Lawley *et al.* [20];

^n^, Horn *et al.* [14] recognized two TraF: the one presenting the best score is pc1438; by colinearity analysis, the other one, i.e. pc1431, seems to be similar to *trsF* of plasmid R391 of *P. rettgeri*;

^o^, annotated as putative TraG by Horn *et al.* [14], BLASTs also with TraG of plasmid F of *E. coli*;

^p^, using a *E*-value cut-off of 0.001, the best BLAST hit is a phage-related protein;

^q^, the 2^nd^ DR is similar to those found at both ends of the GI, with a 14-bp identical sequence and only 3 mismatches out of 17-bp. No other 17-bp DRs with the same conserved 14 nucleotides are present along the UWE25 chromosome. Moreover, since the probability of having a third direct repeat with the same 14-bp is lower than 0.0003, the presence of this homologous direct repeat was probably important during the evolution of this GI, suggesting a possible present or past mobilisation of the proximal 75-kb and/or of the distal 25-kb DNA regions the GI. This hypothesis is strengthened by the presence of an ORF (pc1451) encoding an ATPase involved in DNA repair immediatly flanking this DR;

^r^, this large G+C-rich ORF exhibits a similarity with human NOD protein C-terminus; 5 other homolgous G+C-rich genes are present along the chromosome of UWE25;

^s^, phage-related protein;

^t^, putative DNA-binding protein based on additional BLAST hits;

^u^, putative transposase based on additional BLAST hits: this ORF encodes an homolog of a transposase of *Enterococcus faecium* (score of 74, e-value of 9x10 ^-13^, 50% similarity, 29% identity);

^v^, using a *E*-value cut-off of 0.001, the best BLAST hit is a transposase of *Moraxella* sp. (score=37, *E*-value of 0.16, 67% similarity, 48% identity);

^w^, the next best BLAST hits are transposases of *Pseudomonas aeruginosa* (gamma-proteobacteria).
