# Supplementary figures and images for: A genomic island present along the bacterial chromosome of the Parachlamydiaceae UWE25, an obligate amoebal endosymbiont, encodes a potentially functional F-like conjugative DNA transfer system
Source: BMC Microbiol. 2004 Dec 22;4:48. doi: 10.1186/1471-2180-4-48 (PMC548262; doi:10.1186/1471-2180-4-48)

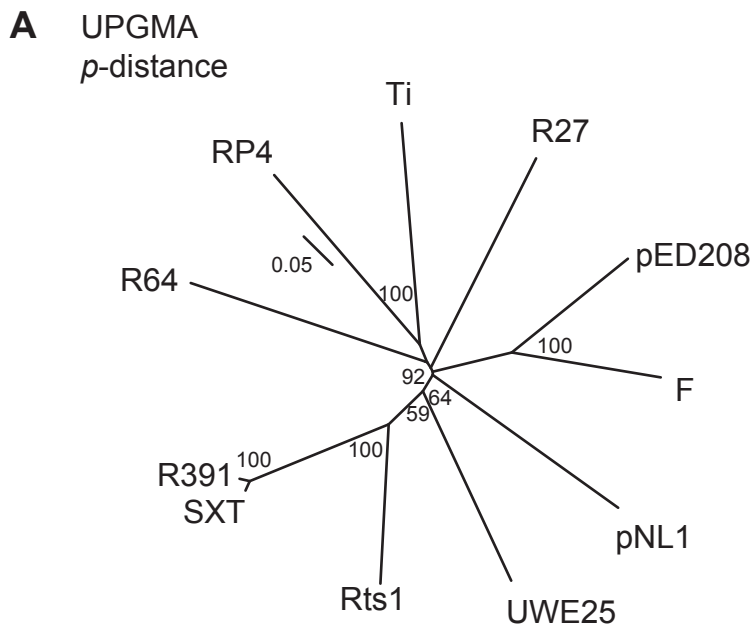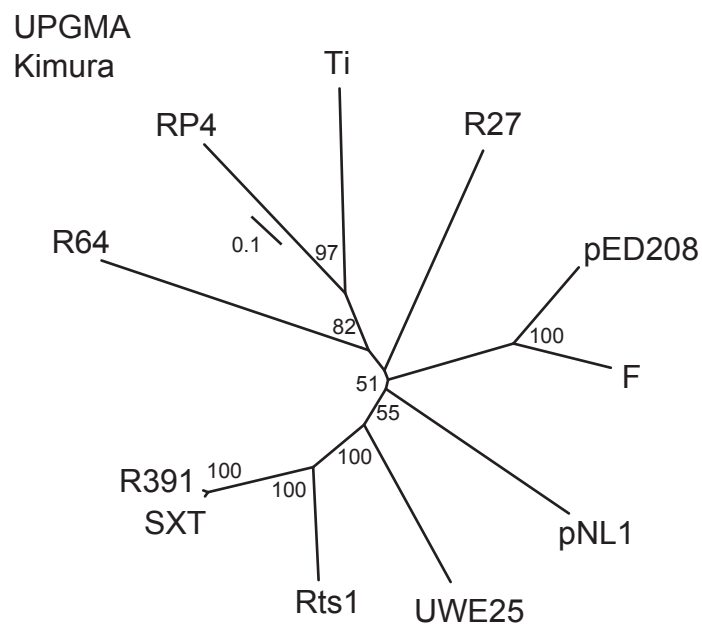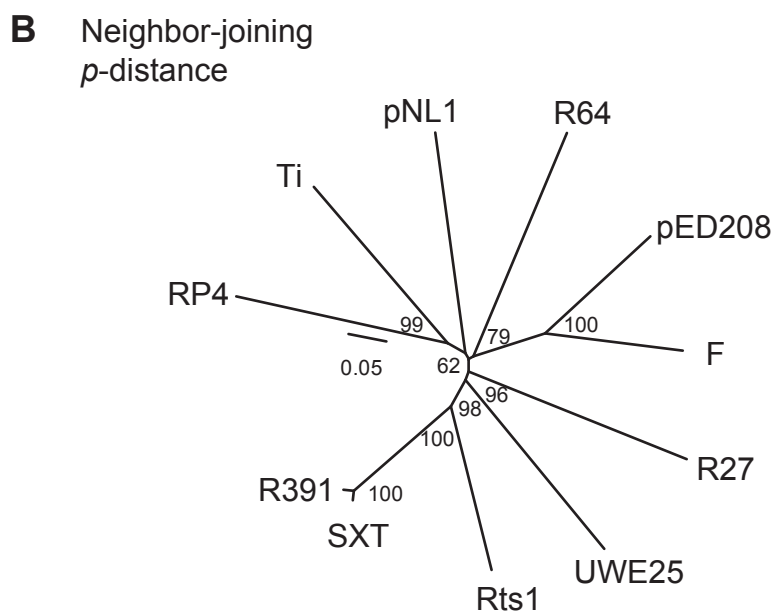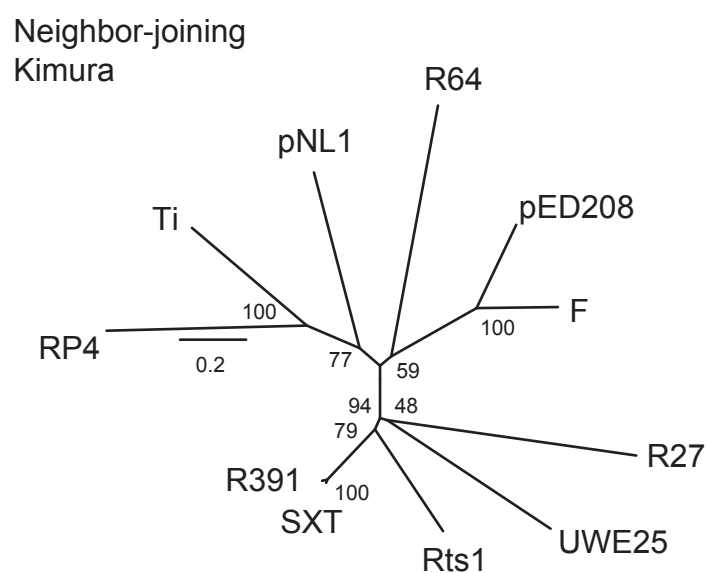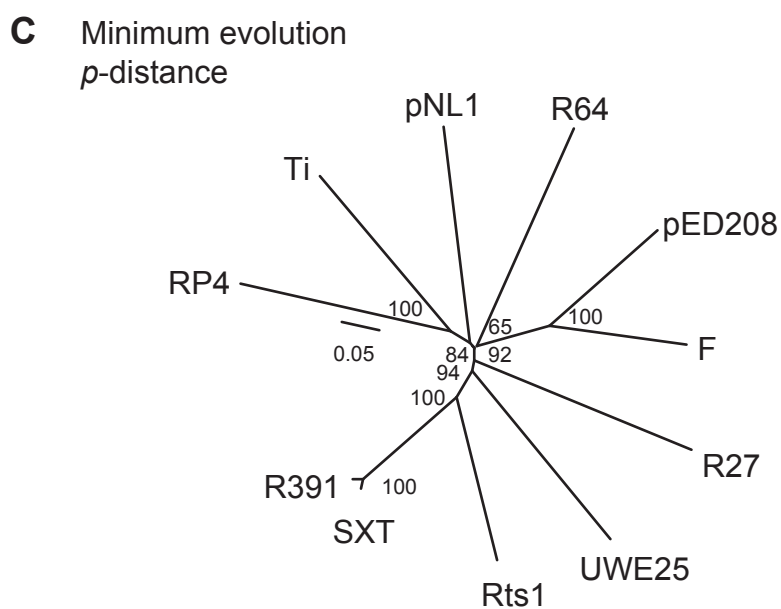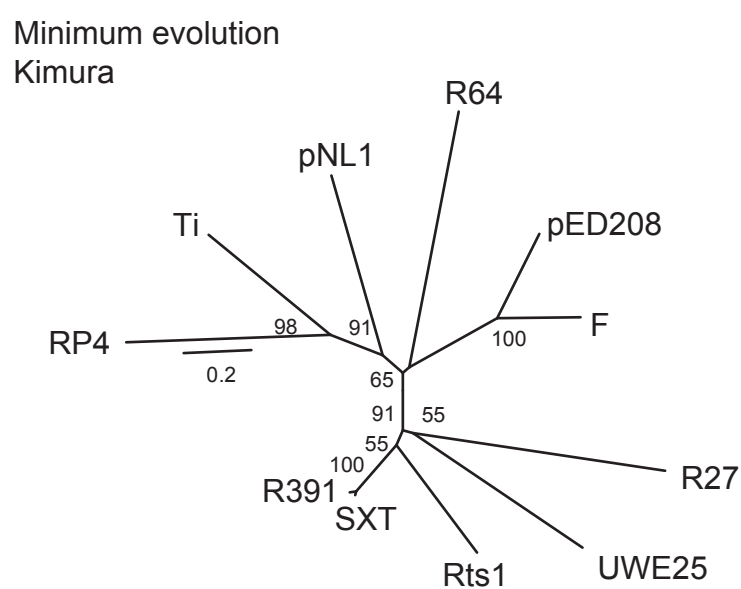

Supplement: Additional File 2 — Supplementary figure. Phylogenetic analyses suggest that the UWE25 tra unit is phylogenetically closely related to F-like DNA conjugative tra operons: (A) UPGMA-, (B) Neighbor-joining-, and (C) minimum evolution-trees comparing p-distances and Kimura corrected p-distances of nucleotide sequences of the concatenated traA, traK, traB, traV, and traC genes (the UPGMA tree comparing the Kimura corrected p-distances of tra genes, shown in Figure 3B, is presented here to facilitate comparison with the other trees). Interestingly, in neighbor-joining and minimum evolution analyses of the p-distances, the tra unit of UWE25 is clustered with tra operons of gamma-proteobacterial F-like plasmids: bootstrap values of 96% and 92%, respectively, support the node separating the concatenated tra genes of UWE25 and RTS1, SXT, R391, three gamma-proteobacterial F-like conjugative plasmids, from their closest relative R27 plasmid. Similarly, in neighbor-joining and minimum evolution analyses of the Kimura corrected p-distances, bootstrap values of 94% and 91%, respectively, support the node separating the concatenated tra genes of the chromosomal UWE25 and the R27 plasmid, another gamma-proteobacterial F-like conjugative plasmid, from those of all other plasmids. [file 1471-2180-4-48-S2.pdf]
